# Inclusion of Individuals With Lived Experiences in the Development of a Digital Intervention for Co-Occurring Depression and Cannabis Use: Mixed Methods Investigation

**DOI:** 10.2196/54751

**Published:** 2024-10-07

**Authors:** Amanda C Collins, Sukanya Bhattacharya, Jenny Y Oh, Abigail Salzhauer, Charles T Taylor, Kate Wolitzky-Taylor, Robin L Aupperle, Alan J Budney, Nicholas C Jacobson

**Affiliations:** 1 Center for Technology and Behavioral Health Geisel School of Medicine Dartmouth College Lebanon, NH United States; 2 Department of Biomedical Data Science Geisel School of Medicine Dartmouth College Hanover, NH United States; 3 Department of Psychiatry University of California San Diego School of Medicine San Diego, CA United States; 4 Department of Psychiatry and Biobehavioral Sciences University of California - Los Angeles Los Angeles, CA United States; 5 Laureate Institute for Brain Research Tulsa, OK United States; 6 Department of Psychiatry Geisel School of Medicine Dartmouth College Hanover, NH United States

**Keywords:** digital intervention, depression, cannabis use, positive affect, formative research, app development

## Abstract

**Background:**

Existing interventions for co-occurring depression and cannabis use often do not treat both disorders simultaneously and can result in higher rates of symptom relapse. Traditional in-person interventions are often difficult to obtain due to financial and time limitations, which may further prevent individuals with co-occurring depression and cannabis use from receiving adequate treatment. Digital interventions can increase the scalability and accessibility for these individuals, but few digital interventions exist to treat both disorders simultaneously. Targeting transdiagnostic processes of these disorders with a digital intervention—specifically positive valence system dysfunction—may yield improved access and outcomes.

**Objective:**

Recent research has highlighted a need for the inclusion of individuals with lived experiences to assist in the co-design of interventions to enhance scalability and relevance of an intervention. Thus, the purpose of this study is to describe the process of eliciting feedback from individuals with elevated depressed symptoms and cannabis use and co-designing a digital intervention, Amplification of Positivity—Cannabis Use Disorder (AMP-C), focused on improving positive valence system dysfunction in these disorders.

**Methods:**

Ten individuals who endorsed moderate to severe depressive symptoms and regular cannabis use (2-3×/week) were recruited online via Meta ads. Using a mixed methods approach, participants completed a 1-hour mixed methods interview over Zoom (Zoom Technologies Inc) where they gave their feedback and suggestions for the development of a mental health app, based on an existing treatment targeting positive valence system dysfunction, for depressive symptoms and cannabis use. The qualitative approach allowed for a broader investigation of participants’ wants and needs regarding the engagement and scalability of AMP-C, and the quantitative approach allowed for specific ratings of intervention components to be potentially included.

**Results:**

Participants perceived the 13 different components of AMP-C as overall helpful (mean 3.9-4.4, SD 0.5-1.1) and interesting (mean 4.0-4.9, SD 0.3-1.1) on a scale from 1 (not at all) to 5 (extremely). They gave qualitative feedback for increasing engagement in the app, including adding a social component, using notifications, and being able to track their symptoms and progress over time.

**Conclusions:**

This study highlights the importance of including individuals with lived experiences in the development of interventions, including digital interventions. This inclusion resulted in valuable feedback and suggestions for improving the proposed digital intervention targeting the positive valence system, AMP-C, to better match the wants and needs of individuals with depressive symptoms and cannabis use.

## Introduction

### Background

Cannabis is one of the most widely used substances worldwide [1,2]. Its impact on mental health has been well-defined, with prior research suggesting genetic [3] and neurobiological [4,5] evidence contributing to an increased risk for depressive symptoms. Cannabis use disorder (CUD) is often highly comorbid with major depressive disorder (MDD) such that the risk for MDD is about 3 times higher in individuals with CUD [6]. In particular, heavy cannabis use and younger age of onset have been associated with higher risk for MDD and depression symptom severity [7-9].

### The Impact of the Positive Valence Systems

The positive valence systems (PVS) are associated with overall reward processing, including how one responds to reward and pleasurable experiences, and dysfunction in the PVS is associated with both MDD and CUD. Reward processing in the PVS is often assessed via 3 pathways: reward responsiveness, reward learning, and reward valuation [10]. Anhedonia, or the loss of interest in pleasure, involves deficits in reward responsiveness and learning and has been found to be elevated in individuals with substance use disorders (SUDs) [11]. Prior work has highlighted the potential role of anhedonia both *causing* drug use and being a *consequence* of drug use. First, studies have shown that baseline anhedonia is related to increased drug use, including cannabis [12]. Second, prior studies indicate that drug use can induce anhedonia [11,13], and findings from a systematic review indicate that anhedonia declines following periods of abstinence, suggesting that drug use may maintain elevated levels of anhedonia [14]. Moreover, studies focusing on other substance use outcomes, including smoking cessation [15] and cocaine use treatment [16], indicate that anhedonia is associated with poorer treatment results, suggesting its clinical relevance for substance use treatment.

The prevalence of anhedonia is particularly elevated within individuals with comorbid SUDs and MDD [17]. For example, individuals without preexisting depressive symptoms who misuse cannabis are more likely to experience anhedonia and suicidal ideation [18]. Chronic cannabis use can cause neurobiological effects including hypodopaminergic anhedonia, increasing the risk for associated depressive symptoms [13]. Indeed, depressive anhedonia may reflect changes in reward processing, including reward hyposensitivity [19]. A corresponding hypersensitivity to drug-related pleasure may contribute to excessive drug usage and relapse, as well as an altered hedonic “set point” that emphasizes drug-related pleasure over other hedonic activities [17]. Thus, anhedonia may serve as a transdiagnostic mechanism between common dimensions for MDD and CUD. For individuals with CUD and MDD, anhedonia may result from difficulties experiencing pleasure from nondrug rewards, reflecting a sensitized reward system [20].

Thus, reward dysfunction in the PVS associated with many SUDs may also play a part in cannabis-depression associations. Individuals with comorbid mood disorder and cannabis use demonstrate reduced reward-related brain activation in striatal regions (in response to nondrug rewards) than those with no cannabis use, suggesting a potential relationship between cannabis use and subsequent changes in reward processing with affective symptoms [21,22]. Moreover, the temporary relief to affective states related to MDD symptoms may reinforce cannabis use over time. For example, some persons may use cannabis to decrease their high negative affect (ie, depressed mood), and others may use cannabis to increase their low positive affect (ie, anhedonia) [23]. Thus, reward constructs within psychopathology, including anhedonia, may contribute to comorbidity between SUDs and MDD and could potentially serve as a transdiagnostic risk factor for these disorders.

### Existing Treatments for Cannabis Use and MDD

Existing treatments for CUD have generally focused on psychotherapeutic treatments including cognitive behavioral therapy (CBT), contingency management, and motivational enhancement therapy [24-26]. Many studies have examined treatments using a combined CBT, contingency management, and motivational enhancement therapy approach, indicating that the combination of all 3 treatments has been most effective in cannabis treatment [27-32]. However, there remains much room for improvement in both magnitude and duration of outcomes and accessibility. Meta-analytic findings have revealed that these therapies are superior to control or waitlist conditions; however, they are not superior to other active control therapies, including treatment as usual [33]. Moreover, it has been hypothesized that withdrawal symptoms, a primary reason for relapse, is related to the endocannabinoid system [34]. Given that this system is implicated within the PVS, it may thus be an important target for treatment of CUD to improve outcomes. Relatedly, previous clinical trials focused on treating cannabis use alone have reported worse outcomes when patients endorse MDD symptoms, compared with those who do not, indicating that the presence of MDD symptoms may contribute to increased cannabis use after the end of treatment [35]. Thus, alternative treatments may be necessary to improve outcomes, particularly for the treatment of co-occurring depression and cannabis use.

Individuals with MDD demonstrate reward dysfunction and a dampened ability to experience positive emotions [36]. Enhancing the PVS, including increasing positive affect and reward functioning, is immeasurably important for mental health and well-being [37-39]. However, traditional therapies, including CBT, often focus on targeting the negative valence systems (NVS), which are responsible for negative emotions (eg, fear) and negative cognitions [40], by restructuring negative thought patterns and schemas [41]. Although second-wave therapies, including behavioral activation, extend beyond traditional CBT to increase pleasurable activities and demonstrate efficacy for MDD [41], treatments focused on exclusively upregulating the PVS with a variety of different activities and skills may be beneficial to target reward dysfunction in the PVS [42-44]. Although novel, PVS-targeted treatments for MDD and anxiety have received extensive support, yielding promising outcomes in reducing symptoms in the short and long term, including anhedonia and depressed mood [43-46]. Treatments targeting the PVS may thus be particularly beneficial to those who do not respond well to traditional therapies such as CBT, which place a greater emphasis on reducing negative affect than increasing positive affect [37,38,44-47]. Indeed, existing research with PVS-targeted treatments has shown that they are effective in treating MDD and appear to be effective at improving PVS outcomes (eg, positive affect and well-being) while also reducing NVS outcomes (eg, negative affect, depressive symptoms, and anxiety symptoms) [46]. Thus, these treatments may be well suited to treat MDD given that they can improve dysfunction in both the PVS and the NVS, despite the explicit focus on the PVS, and the implementation of a digital intervention that targets the PVS may help improve inaccessibility concerns (for traditional in-person therapies) for individuals with MDD.

### Amplification of Positivity Treatment

In one specific PVS-targeted treatment, Amplification of Positivity (AMP), participants with clinically high anxiety and depression learned skills to enhance the PVS with respect to the following topics: identifying and amplifying positive experiences, gratitude, acts of kindness, planning and taking part in pleasurable, engaging and meaningful activities, identifying and exemplifying personal values, imagining and actualizing positive thoughts about their futures, living like they will leave their area soon, and developing a personalized positive activity plan [44]. Compared with the control group, the treatment group reported reduced depressive symptoms and increased positive affect up to 6 months after completion of treatment (η^2^_p_=0.22-0.39). Considering the results of this intervention, among others investigating the effects of this intervention, AMP as an in-person treatment appears to demonstrate significant efficacy in improving depressive and positive affect outcomes [43,44,48,49].

Although MDD co-occurs with SUDs, most treatments address the disorders separately, often focusing on one disorder over the other [50]. Research suggests that integrative treatments may be more effective than parallel treatment for MDD and SUDs [26,50-53]. Because the PVS is impacted in both MDD and SUDs, it may be an important transdiagnostic treatment target [43,54,55]. Recently, AMP has been modified to treat co-occurring depression and substance use, and an initial pilot study implemented this modified, in-person AMP protocol to simultaneously treat alcohol use disorder, MDD, and anxiety [43]. Findings indicated significant improvement in the frequency of alcohol use, positive and negative affect, and overall functioning. Moreover, although not the main focus of treatment, some participants reported a decrease in their cannabis use, indicating that AMP may be suitable for targeting other SUDs, including CUD [43].

### Digital Interventions

Although AMP has demonstrated an ability to improve depressive symptoms and substance use, prior trials have used a traditional format (eg, in-person individual therapy), and in-person treatments are often not scalable to the larger population [41]. As such, digital interventions are a suitable option to increase scalability of a treatment and are more cost-effective than traditional therapy [56]. Digital interventions are becoming increasingly accessible to those who may experience financial, geographic, temporal, or stigma-based barriers to mental health treatment [57,58]. Digital interventions (eg, on a smartphone app) allow individuals to complete a treatment at their own pace and when and where they are most comfortable [59]. In addition, implementing a personalized digital intervention may help relieve the burden on mental health professionals due to the paucity of mental health treatment providers [60]. Despite the promise in digital interventions improving outcomes, there may be some participant concerns regarding digital interventions (eg, engagement) that need to be addressed.

### This Study

Recent research has emphasized the role of including individuals with lived experiences (commonly referred to as “experts by experience” [EBEs] from here on out) in the development and evaluation of psychological interventions [61]. This is particularly important to (1) determine what skills or components EBEs would find helpful or valuable to include in an intervention and (2) provide ideas of what strategies or facilitators could be used to improve engagement in and completion of the intervention. Given this, the present study sought to use a co-design approach with EBEs to better understand the wants and needs of individuals with co-occurring depressive symptoms and cannabis use, with the overall aim to inform creation of a digital intervention that would be more accessible and adaptable to the target population.

Given the potential role of PVS dysfunction in the development, maintenance, and comorbidity of MDD and CUD, and the absence of integrative treatments that focus on the PVS in co-occurring MDD and CUD, the overall purpose of this study was to develop a digital intervention focused on the PVS. As such, we included EBEs, or individuals with problematic depressive symptoms and cannabis use, to help design the digital intervention, AMP-C. This study focuses on the first interview in a larger project with an iterative and longitudinal design to the development of AMP-C. Specifically, in a mixed methods interview, participants provided feedback on their preferred duration, frequency, and format of sessions if they were to engage in the therapy as well as the components or topics, focusing on the PVS, that they would find helpful for sessions. Thus, this manuscript discusses the process of interacting with persons with lived experiences and involving their feedback in the initial development of AMP-C. We also provide initial results of participants’ feedback and ratings of components to be potentially included in the intervention, which is based off of prior work [42], with the goal of using participants’ feedback from this first interview to tailor the development of AMP-C during this iterative project.

## Methods

### Ethical Considerations

This study was approved by the Dartmouth College Institutional Review Board (the Committee for the Protection of Human Subjects [STUDY00032686]).

### Recruitment

**Figure 1:**
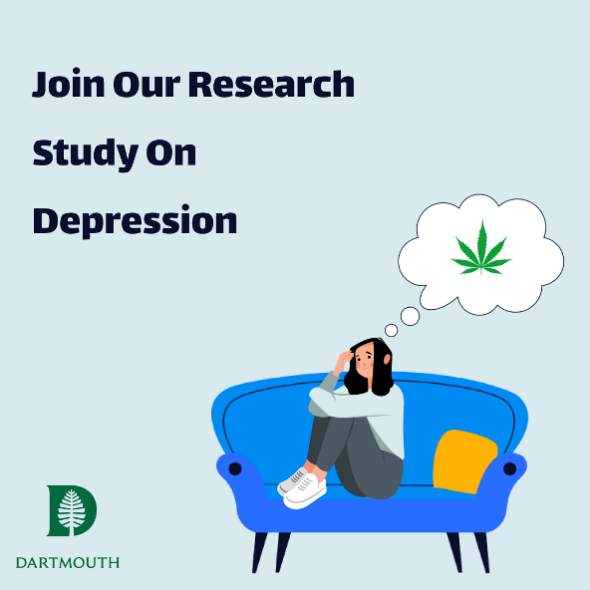
Meta ad presented to potential participants on Facebook and Instagram. This is the advertisement presented to targeted individuals on Facebook and Instagram to recruit them for a study on depression.

The methods and results for this study, including the procedures for the mixed methods interview, are reported following the COREQ (COnsolidated criteria for REporting Qualitative research) checklist, and the checklist is available in Multimedia Appendix 1 [62]. Participants from the United States were recruited via meta advertisements and received targeted ads on Facebook and Instagram (Figure 1).

Upon clicking on the ad, participants were redirected to the screener survey on Qualtrics. After reading an informed consent, participants completed several self-report measures, including the Patient Health Questionnaire-9 (PHQ-9) and the Cannabis Use Disorder Identification Test—Revised (CUDIT-R).

If participants endorsed a score greater than 0 on item 9 on the PHQ-9 (ie, the suicidality item), then participants were given the Columbia-Suicide Severity Rating Scale (C-SSRS), which is the gold standard in comprehensive suicide risk assessment [63] (see section “Screening Measure” for more detail on these measures). After completing the self-report measures, participants were asked “Are you interested in receiving treatment for your depressive symptoms or cannabis use?” Participants who responded “Yes” were prompted to enter their demographic information, including name. Finally, participants were shown a list of national resources (eg, SAMHSA Suicide & Crisis Lifeline) given the sensitive nature of some of the questions asked (eg, suicidality).

#### Screening Measures

The PHQ-9 is a 9-item self-report measure that assesses depressive symptoms over the previous 2 weeks [64,65]. The PHQ-9 is commonly used for screening depressive symptom severity, and scores of 10 or above on the PHQ-9 represent at least moderate symptom severity and likely MDD diagnosis [64]. The CUDIT-R is an 8-item self-report measure that assesses cannabis consumption, abuse, dependence, and psychological symptoms related to cannabis use over the previous 6 months, with scores of ≥12 indicating a probable diagnosis of CUD [66]. The C-SSRS is an efficient, reliable, and valid self-report measure that is commonly used in clinical and research settings to screen for suicidal thoughts and behaviors, including suicidal ideation, intent, plan, or previous attempts [63]. Moreover, the C-SSRS has been found to better detect active suicidality than item 9 on the PHQ-9 (ie, the suicide item), which has been shown to produce higher false-positive rates [67].

#### Inclusion Criteria

**Figure 2:**
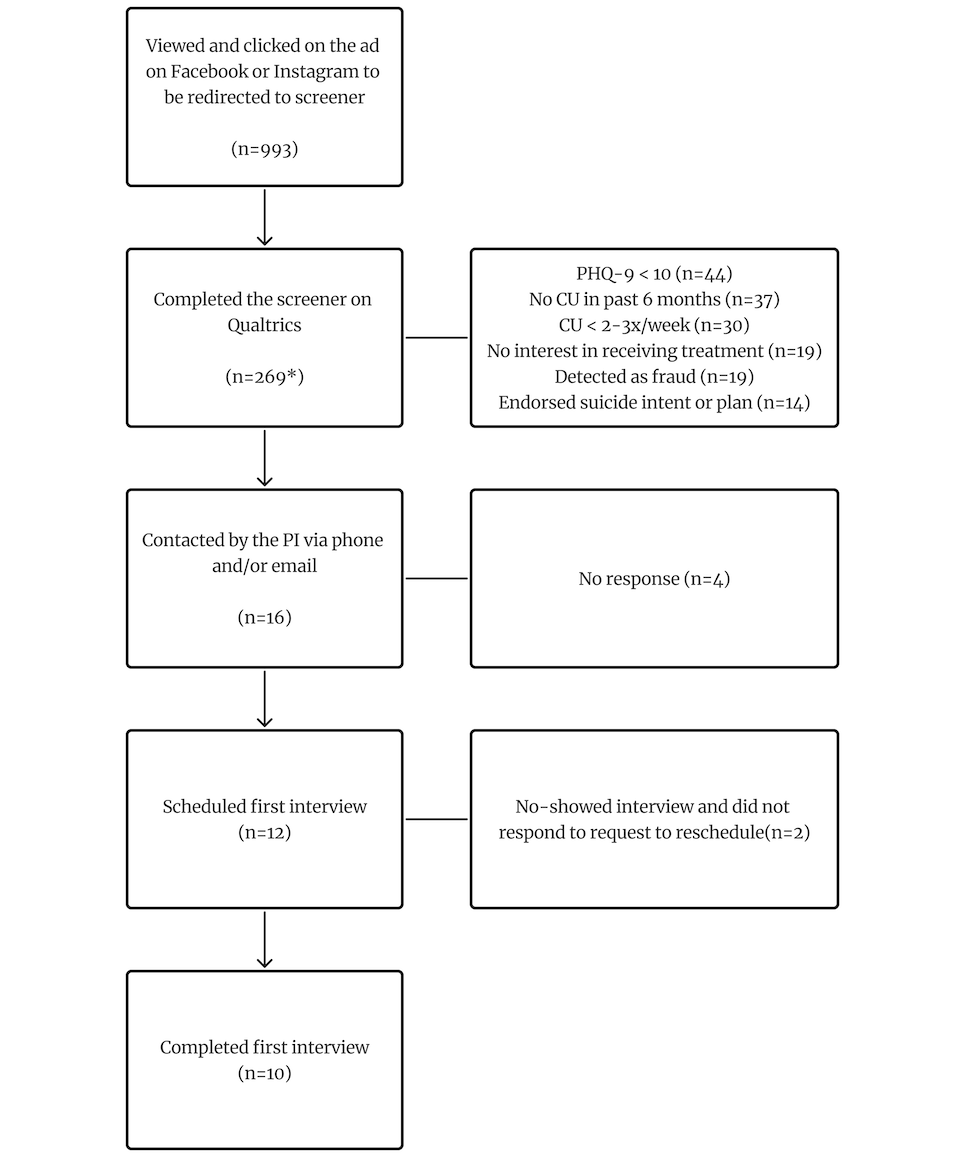
Recruitment and screening process. This is the flow diagram for the recruitment process and reasons for eligibility, with 10 participants meeting criteria for the study and completing the first interview. *Indicates that some participants who completed the screener on Qualtrics may not have met criteria to be contacted due to several reasons (eg, PHQ-9 <10 and no CU in the past 6 months). CU: cannabis use; PHQ-9: Patient Health Questionnaire-9; PI: principal investigator.

Participants were contacted for inclusion in the study if they met the following criteria: (1) interested in receiving treatment for depressive symptoms or cannabis, (2) moderate depression as measured by the PHQ-9 (ie, PHQ-9 ≥10), (3) regular cannabis use (ie, at least 2-3 times a week), and (4) between the ages of 18 and 65 years. Participants were excluded if they endorsed suicidal intent or plan, as indicated on the C-SSRS. To ensure high data quality on the screener, we implemented several verification checks with Qualtrics’ fraud detection settings, including “prevent multiple submission,” “bot detection,” “security scan monitor,” and “relevantID.” Participants who had their survey response flagged for a fraudulent response were not contacted to enroll in the study. After filtering participant responses for inclusion and exclusion criteria, 96 participants were eligible for this study. Participants who met criteria for the study were contacted via phone or email to be given more information about the study and be invited to participate (see Figure 2 for recruitment overview). Moreover, participants were contacted based on their self-reported demographics (eg, gender, race, and ethnicity) in an effort to recruit diverse participants that matched the demographics of the population within the United States at the time of recruitment.

### Participants

Ten participants enrolled in the study after providing a signed informed consent. This sample size is comparable with those in existing studies that have implemented a mixed methods approach to collect both quantitative and qualitative responses in developing digital interventions [68-70]. These participants ranged widely in their overall demographics, including symptomatology, gender, age, race, and ethnicity (see Table 1 for a breakdown of participant demographics).

**Table 1:** Participant demographics (N=10).

Demographics				Values
Age (years), mean (SD)				31.6 (7.69)
**Gender, n (%)**				
	Men		5 (50)	
	Women		4 (40)	
	Transgender man		1 (10)	
**Race, n (%)**				
		American Indian/Alaska Native	1 (10)	
		Asian	1 (10)	
		Black or African American	2 (20)	
		White	5 (50)	
		Self-describe (“Hispanic”)	1 (10)	
**Ethnicity, n (%)**				
		Non-Hispanic	8 (80)	
		Hispanic	2 (20)	

On average, participants reported moderately severe levels of depressive symptoms on the PHQ-9 (mean 14.9, SD 4.36; range: 10-24) and a probable diagnosis of CUD on the CUDIT-R (mean 16.3, SD 4.68; range: 11-24). Also, 60% (6/10) of the participants reported a history of psychological treatment for various mental health reasons, including depression, anxiety, and posttraumatic stress disorder. Moreover, 50% (5/10) of the participants reported a history of psychotropic medications. Several participants (4/10, 40%) reported using cannabis for multiple reasons including as a coping mechanism, for relaxation, and for somatic symptoms, while 10% (1/10) of the participants reported using cannabis as a method to quit use of another substance. Also, 50% (5/10) of the participants reported using cannabis multiple times daily.

### Mixed Methods Interview

The purpose of the first mixed methods interview, which was created following the IDEAS (Integrate, Design, Assess, and Share) framework [70], was to gather initial information regarding participants’ preferences for a digital intervention to target the PVS for co-occurring MDD and CUD. They were informed that the researchers were creating a digital intervention for this purpose and would like to create the digital intervention based on the feedback of individuals with lived experiences. These questions for the current interview were developed in an iterative process. First, an undergraduate student (AS) used examples from prior work to guide the development of the initial interview questions [43,44,70-74]. Then, the questions were revised by other members of the research team (ACC, AS, JYO, and SB). The resulting interview questions were further revised by the principal investigators (ACC and NCJ), both of whom agreed upon the final version to be used in the interview (see Multimedia Appendix 1 for a final version of the interview questions).

Two of the interviews were conducted by the principal investigator (ACC), who had a doctoral degree in clinical psychology, with the undergraduate research assistants (RAs) observing the interviews, and the 8 other interviews were conducted alone by these undergraduate RAs (AS, JYO, and SB). The interviewers all identified as women with different racial backgrounds (ie, Asian, Indian, and White), and their ages were all within the range of 20-30 years at the time of the interview. The interviewers all had training in study design and methodology, and training for the qualitative interviews, specifically, occurred through formal meetings with the study team. There was no established relationship between the interviewers and participants prior to the beginning of interview, and all participants were informed that they would be completing the interview with either the principal investigator or the trained undergraduate RA.

The interview lasted approximately 1 hour over Zoom (Zoom Technologies Inc), and participants were compensated US $50 for their time. Participants were asked a range of questions to better understand their experience with mental health, cannabis use, prior smartphone use, prior experience with mental health apps, and preferences about app-based mental health interventions. First, interviewers asked the participants additional demographic- and cannabis use–related questions. Participants were also asked about their use history with mental health apps, 60% (6/10) of whom had used mental health apps in the past. They were also asked about their preferences for future engagement with such apps.

After gathering general information about their mental health history and apps, participants were presented with 13 potential intervention components to be included in the app, which consisted of psychoeducation and learning new skills. Amplification of Positivity for Alcohol Use Disorder (AMP-A) is a 12-session in-person therapy for the treatment of substance use [43], which was adapted from the AMP treatment [44]. Thus, the 13 potential components were adapted from AMP-A with the exception of the “reward devaluation” component (see Table 2 for a further description of each component). The latter component focused on providing participants with information about how and why some individuals devalue positivity, and it was added with the intent to increase buy-in for the intervention given that individuals who devalue positivity may be more likely to drop out of treatments focusing on upregulating the PVS [75]. For each of these potential components, participants first provided broad qualitative feedback on their thoughts and opinions. Then, they responded to questions regarding how interesting and how helpful they thought the given component would be in reducing their depressive symptoms and cannabis use on a 5-point Likert scale ranging from 1 (not at all) to 5 (extremely). After each of these questions, participants were given the opportunity to provide any additional thoughts that they had not yet expressed.

**Table 2:** Description of components.

Component	Summary of component
Psychoeducation	The link between depression and cannabis use, including how they can positively and negatively influence each other.
Emotions	Impact of positive and negative emotions on thinking, behavior, and overall mood, including how emotions can relate to depression and cannabis use.
Reward devaluation	Difficulties of engaging with positive emotions due to avoidance or devaluation, including how one’s views of positivity impact their depressive symptoms and cannabis use.
Noticing and scheduling	Noticing and scheduling positive events that do not include cannabis to increase one’s daily frequency of experiencing positive emotions.
Capitalizing	Skills to prolong, amplify, or intensify positive emotions.
Gratitude	Reflecting on things one is grateful for or showing appreciation for the different people or things in your life can improve positive emotions.
Acts of kindness	Engaging in different acts of kindness, including for family, friends, and strangers, can benefit one’s well-being and positive emotions.
Three paths to positivity	Implementation of pleasurable, engaging, and meaningful activities not involving cannabis to improve positive mood, depressive symptoms, and cannabis use.
Values and strengths	Identifying values and strengths and how engaging in activities that are in line with these can improve one’s positive views about oneself and their world.
Optimism	The benefits and importance of optimism and how cultivating optimism can improve one’s mood.
Social relationships	The importance of social relationships for mental health and well-being and how identifying and implementing positive activities for others can make themselves and someone else happier.
Last in your area	“Seizing the day” by living this month like it will be the last in one’s area to engage in value-aligned activities in one’s area.
Treatment plan	Developing an individualized treatment plan based on the skills in the intervention so that individuals can continue to experience sustainable gains in positive emotions and prevent relapse of cannabis use and depressive symptoms

During their interview, researchers described potential educational components to be included in the app prototype. Participants were asked questions about the extent to which they believed the components would help reduce their cannabis use and depressive symptoms as well as how interesting they found the components. They were also given the opportunity to provide more general, qualitative feedback on each of the 13 components.

### Quantitative Data Analysis

Given the small sample size of this study, we largely focused on descriptives of the quantitative data from the interview. Specifically, we derived the percentages of each endorsed item regarding the app format (Table 3) and calculated the mean and SD of the Amplification of Positivity—Cannabis Use Disorder (AMP-C) component ratings (Table 4). In addition, and despite the small sample size, we wanted to investigate whether there were differences in how participants rated each component (ie, sum of helpfulness and interesting). Thus, we conducted independent *t* tests to compare the ratings of each component against each other (eg, psychoeducation vs emotions). Although conducting multiple comparisons runs the risk of inflating type I error, we wanted to illustrate perceived differences in each rating with our preliminary data of 10 persons. We conducted post hoc power analyses in R using the function *power.t.test*, and results indicated that all of the *t* tests were adequately powered (>90%).

**Table 3:** Engagement in app.

Items	Responses
How often would you want to engage with the app?	6 (daily), 60% 2 (2×/d), 20% 1 (2-3×/wk), 10% 1 (prn), 10%
How many minutes would you like to engage with each component at a time?	2 (<10 minutes), 20% 5 (10-15 minutes), 50% 3 (>15 minutes), 30%
How often would you want to learn a new skill through the app (eg, every day, once a week, etc)?	3 (daily), 30% 1 (2-3×/wk), 10% 4 (weekly), 40% 2 (unspecified^a^), 20%
Would you find it helpful to have reminders/methods to keep you accountable for participating in a mental health app?	10 (yes), 100% 0 (no), 0%
What kind of reminders/methods would be helpful?	2 (push notifications), 20% 1 (text), 10% 1 (rewards), 10% 6 (>1), 60%
Would you prefer to be able to use it more passively (ie, reading things while sitting down/watching a video) or actively (ie, a breathing exercise or guided meditation/mindfulness)?	0 (passively), 0% 5 (actively), 50% 5 (both), 50%
Would you like to have tasks to complete in between content to help practice your skills? These could include, for example, answering short questions about your skill practices.	10 (yes), 100% 0 (no), 0%
Would you like to be able to track your symptoms over time in the app?	10 (yes), 100% 0 (no), 0%
How would you like the content presented to you?	1 (everything at once), 10% 1 (w/ recommendations), 10% 8 (structured), 80%
Would you be interested in receiving bonus or advanced content?	10 (yes), 100% 0 (no), 0%

^a^“Unspecified” refers to participants listing several different frequencies in which they would engage with the app. For example, one participant stated “daily or at least 3-4×/week.”

**Table 4:** Summary of component ratings.

Component	Helpfulness, mean (SD)	Interesting, mean (SD)	Sum of helpfulness and interesting^a^
Psychoeducation	3.9 (0.88)	4.3 (0.82)	8.2
Emotions	4.1 (0.74)	4.6 (0.52)	8.7
Reward Devaluation	3.9 (0.99)	4.2 (0.79)	8.1
Noticing and Scheduling	4.4 (0.7)	4.6 (0.52)	9
Capitalizing	4.5 (0.53)	4.9 (0.32)	9.4
Gratitude	4.3 (0.67)	4.5 (0.71)	8.8
Three Paths to Positivity	4.3 (0.82)	4.4 (0.84)	8.7
Acts of Kindness	4 (0.82)	4.2 (0.92)	8.2
Treatment Plan	4.4 (0.52)	4.4 (0.52)	8.8
Values and Strengths	3.9 (0.99)	4 (0.82)	7.9
Optimism	4 (1.05)	4 (1.05)	8
Social Relationships	4.1 (0.99)	4.1 (0.74)	8.2
Like it’s Your Last Time in the Area	4.2 (0.79)	4 (0.82)	8.2

^a^Perceived Helpfulness and Interesting were rated from 1 (not at all) to 5 (extremely).

### Qualitative Data Analysis

We used thematic coding analysis with a bottom-up approach to analyze the qualitative data from the interview. All interviews were audio-recorded via and transcribed using the built-in software with Zoom. Interviewers also made notes during the interviews to summarize participants’ responses. One author (ACC) reviewed the transcriptions and manually edited the transcripts where there were discrepancies (ie, some words were coded incorrectly). Then, another author (AS) reviewed the transcripts and identified a list of codes: (1) social, (2) mental health support and resources, (3) education, (4) symptom tracking and progress, (5) journaling, (6) notifications, and (7) harm reduction of cannabis. Two authors (ACC and JYO) then independently coded each transcript with the 7 codes selected in the initial review of the transcripts. After coding, interrater agreement was coded, and any discrepancies were reviewed by both authors. The interview transcripts were all coded and analyzed using ATLAS.ti (ATLAS.ti Scientific Software Development GmbH), which is a commonly used software for thematic data analysis. Participants did not receive their respective transcripts or the findings from the qualitative analyses.

## Results

### Quantitative Results: Format of Digital Intervention and App

All participants reported that they would be able to incorporate a mental health app into their current daily routines (Table 3). However, participants had varying views on what kinds of features might be conducive to engaging with the app in a meaningful and useful way. Participants also indicated that customizing, to a degree, the time at which they could use the app was important: 2 participants specified that they would like to use the app in the mornings, while 4 participants reported that they would likely interact with the app at night. Some participants expressed a preference for features that promoted engagement (eg, “Would like to have interactive activities in the morning and night to keep me engaged in the app, like journal prompts”) and that they would like to be reminded to check in with the app (eg, “Would be easier to engage with push notifications to remind me to check in”). However, one participant specified that they would not like to have “too many” notifications and would thus appreciate the ability to customize notification type and frequency.

### Intervention Component Ratings

After participants previewed potential components, they rated the most interesting and helpful components to be Capitalizing, Noticing and Scheduling, Treatment Plan, and Gratitude, respectively (Table 4). The least interesting and helpful components rated were Values and Strengths, Optimism, and Reward Devaluation, respectively. However, it is worth noting that most components were rated extremely helpful and very interesting, with values of at least 4 on a Likert scale of 1 (not at all) to 5 (extremely).

Results from a series of *t* tests revealed that participants rated Capitalizing highest, and this rating was significantly higher than Psychoeducation (*t*_9_=2.714; *P*=.02; 95% CI 0.200-2.200), Emotions (*t*_9_=2.333; *P*=.045; 95% CI 0.021-1.379), Reward Devaluation (*t*_9_=2.512; *P*=.033; 95% CI 0.120-2.471), Values and Strengths (*t*_9_=3.309; *P*=.009; 95% CI 0.474-2.526), and Like it’s Your Last Time in the Area (*t*_9_=2.714; *P*=.02; 95% CI 0.200-2.200). Thus, these findings provide preliminary evidence that persons with depressive symptoms and cannabis use may believe that strategies to increase their experience of positive emotions (eg, savoring) would be the most interesting and helpful for a digital intervention.

### Qualitative Results

The thematic coding analysis provided insight into what features participants thought would increase their interest and engagement in the digital intervention. Several key themes were identified, including journaling with prompts, notifications, checking in on symptoms and mood throughout the day, chatting with an artificial intelligence therapist and other app users, access to local resources in the app, learning new information about depression and cannabis with each use, and learning more about how to effectively use cannabis with a harm reduction approach (see Table 5 for major themes). Of note, participants’ responses regarding app engagement and suggestions came from open-ended questions, so not every participant was asked specifically about each theme (eg, about a social component). Moreover, participants also noted the importance of establishing a consistent routine of app use and communicating with others on the app in order to reap the most benefits from the app. Specifically, participants mentioned that socializing with others in similar positions would be beneficial in reducing stigma and providing and receiving support when necessary. Overall, the interrater simple percent agreement was 89.9%, and the Krippendorff a value was 0.950. Each theme or semantic domain demonstrated a satisfactory level of agreement above 0.80 regarding the suggested cutoffs for Krippendorff a [76].

**Table 5:** Common themes of participants’ feedback on engagement.

Theme	Examples of participant feedback	Simple percent agreement (%)/Krippendorff a
Social	“A social aspect is needed to relate with other people and help with more than mental health.” “Past experiences of others … ability to meet people locally, comment board.”	89.4/0.942
Mental Health Support and Resources	“Local communities to explore, peer to peer networks (sponsor).”	75.5/0.859
Education	“I’m already on the phone most of the day anyways, so it would be no problem for me to take 15-20 minutes to pull out the app to check articles on depression and cannabis or scroll through some coping skills.”	93.5/0.963
Symptom Tracking and Progress	“I think, having like a metric thing that shows you your symptoms, [to] keep track of like, did you have more depressive symptoms today? Did you smoke more? Did you smoke less? I think that would definitely be helpful to see, especially if people can find a correlation between when they feel more depressed.”	87.8/0.934
Journaling	“[I] struggle with keeping a journal and writing things down, so having an interactive thing like a journal might be helpful.”	86.2/0.924
Notifications	“Having a push notification to remind me to check in would be helpful.”	86.9/0.929
Harm Reduction of Cannabis	“Having access to both ways to cut back on smoking and effectively use smoking as it has been both effective and ineffective.”	72.9/0.841

#### Social Component

Three participants expressed wanting a social component of the intervention, including the option to connect with others experiencing similar symptoms for support. Some noted that social connection would help them combat the stigma associated with substance use and depression, which can often feel isolating, while others primarily wanted to hear about others’ experiences. Additional suggestions included local communities to explore, peer-to-peer networks, ability to meet people locally, comment boards, and inspiration sections (eg, a phrase of the day).

#### Psychoeducation and Harm Reduction

Two participants requested educational components, so that they could develop a better understanding of CUD and depression. For example, one participant wanted to understand what happens if you mix cannabis and medications. Relatedly, 2 participants expressed wanting to reduce the potential harm of cannabis use. Whereas participants had different goals—one wanted to learn ways to cut back on smoking cannabis and effectively use smoking as a coping skill while the other wanted a program that helps with stopping cannabis use completely—participants wanted to ensure that they could use cannabis in a safer and healthier way if at all.

#### Support, Resources, and Symptom Tracking

One participant noted that it would be helpful to have a therapist or provider to chat with for clinical advice. Similarly, 3 participants suggested that there be general mental health support and resources available within the app. Two participants also suggested a tracking system, where they could reflect on their days to track their mood, progress, and cannabis consumption, which may also help identify triggers behind cannabis use. One participant specifically noted that logging these data could help identify potential triggers, and another participant wanted ideas or tips to help cope with their reported feelings. Collectively, participants felt that being able to track and write or journal about the symptoms and stressors would be helpful for monitoring changes in their symptoms and daily life.

#### App Notifications

Three participants also discussed push notifications; however, these responses were mixed. One participant stated that the app should help users to reach out when they need the support, but they specifically asked not to be bombarded with alerts and notifications. Other participants wanted notifications to help them get into the habit of using the app by using it at the same time each day. Some participants suggested that there be daily engagement exercises, which might include questions of the day and something to turn to on a bad day. Finally, one participant suggested that gamification be incorporated into the app to increase user motivation. Ultimately, the participants seemed to value interpersonal connection, having a mood and cannabis tracker, journaling about their days, establishing a routine to engage with the app, and learning more about cannabis use and their mood.

## Discussion

### Principal Findings

In this study, we sought to include the feedback from individuals with lived experiences of depressive symptoms and cannabis use to help develop a digital intervention focused on PVS dysfunction, AMP-C. Their feedback during the mixed methods interview yielded important information regarding ways to improve the intervention components, other features to include in the app, and format of the app. Participants rated each of the 13 components as overall positive; however, some components emerged as more engaging and interesting than others. These included learning more about (1) the role of positive emotions and negative in depression and cannabis use, (2) noticing and scheduling positive events not related to cannabis use, and (3) capitalizing on positive events not related to cannabis use. Indeed, these 3 intervention components are crucial in enhancing the PVS and orienting participants’ attention toward nondrug rewards and positive information [43,44]. Whereas the other component is largely related to learning new ways about how to cultivate positive emotions, these 3 components focus on how to initially track and prolong the positive emotions that one does experience *without* using cannabis. Moreover, participants varied widely on their goals for cannabis use, with some citing abstinence and others citing a harm reduction approach, the latter of which was implemented within AMP-A for treating alcohol use.

Taken together, the findings from this initial interview led us to several conclusions of how to revise the AMP-C prototype moving forward. First, participants varied in their ratings of each intervention component, but they were in unanimous agreement that they wanted bonus components within the intervention. Thus, we concluded that it would be beneficial to not only include a few core intervention components but also have a “bonus” component that would be available once participants completed the core components. As such, we revised the intervention structure to have 6 core components with 2-3 sessions in each component. In addition, we created a bonus component with five sessions, including many of those that were rated the lowest by participants: (1) values, (2) strengths, (3) optimism, (4) last in your area, and (5) social relationships. Second, participants had different goals for their cannabis use. Thus, we adapted the intervention to ensure that the goals component emphasized a personalized approach such that participants could set individual goals for their cannabis use, ranging from stopping their use to decreasing the frequency of use (ie, to a certain number of days per week, only in social situations, etc). Third, there were several features that participants wanted in the app, including local resources. Thus, we included a button within the app that participants could press if they were experiencing a mental health crisis or wanted to find local support resources.

### Limitations

Although the current findings provide important insight into the device usage, digital app preferences, and views about intervention components for individuals with depressive symptoms and cannabis use, there are some limitations to this study. First, to focus on the quality of the information collected, rather than quantity, we recruited only 10 participants for the interview. Moreover, we recruited these participants exclusively on social media (eg, Facebook and Instagram). Taken together, it is possible that the current sample is not representative of the larger population (and subpopulation of those with co-occurring depression and cannabis use). We used a stratified sample design during recruitment to collect a heterogeneous sample in regarding race, ethnicity, gender, age, geographical location, depressive symptoms, and cannabis use, which helped increase the representativeness of the sample. In addition, we did not include individuals with formal diagnoses of MDD and CUD. Rather, we used cutoff scores on the PHQ-9 and the CUDIT-R to assess for moderate depressive symptoms and probably CUD, respectively, to reduce burden on participants and the researchers. The severity of symptoms may impact how one responds to a digital intervention [77], so it is important for future work to gather feedback from persons with formal diagnoses (and more severe symptoms) when developing digital interventions. Finally, although we consider use of both quantitative and qualitative methodology as a strength in this study, some of the identified themes from the qualitative coding did contain some overlap. For example, participants’ quotes relating to wanting to talk to other users and meet them locally (eg, in a support group) could be coded in both the social and resource themes.

### Comparison With Prior Work

Given that digital interventions can be heterogeneous in both content and structure, it is becoming increasingly more common to include EBEs in the development of digital interventions. For example, 1 study included individuals with frequent cannabis use to help develop supportive messages to be included in a digital intervention for cannabis use [68]. Using an iterative process, EBEs provided feedback on the supportive messages, including whether the messages were lengthy, unclear, triggering, or not helpful. This feedback was used to revise the supportive messages, and the EBEs gave higher ratings regarding the usefulness of the supportive messages after revisions. These final supportive messages were then included in the digital intervention being developed [68]. The process of incorporating EBEs into the development of digital interventions is becoming more common [78,79]; however, there is still a need to continue to involve EBEs to better understand their needs and improve interventions and mental health outcomes. As such, this work is the first to implement a co-design process with EBEs to develop a digital intervention for co-occurring MDD and CUD. This is particularly important given that existing in-person treatments are often not effective in the long term for improving symptoms, and many of these were not created with individuals with EBEs. As such, gathering feedback from individuals with EBEs will aid in developing a digital intervention (ie, AMP-C) that is likely both effective and enduring, providing the opportunity for AMP-C to be used (1) as an adjunct to in-person therapy, (2) while a person is on waitlist for in-person therapy, (3) during the maintenance phase after discontinuation of therapy, or (4) for those who are not able to receive in-person therapy due to many of the aforementioned barriers and are seeking a self-guided option.

### Future Directions

This study served as the first, formative step of a 3-step project, and future directions of this work include conducting 2 more mixed methods interviews with the same 10 participants. The second interview will include asking more questions regarding their feedback about the app and digital intervention, designing their own prototype of a digital intervention, and giving feedback about the mock prototypes that were created based on their responses in the first interview. In the third interview, participants will test AMP-C after it is developed and provide more feedback, including the feasibility, acceptability, and usability of the digital intervention. This process will result in a deployable digital intervention focused on PVS dysfunction for co-occurring depression and cannabis use. Future research will then aim to investigate the efficacy of AMP-C in a randomized controlled trial.

### Conclusions

This study highlights the importance of including individuals with lived experiences in the development of interventions, including digital interventions. Specifically, given that this is the first known digital intervention that aims to target PVS dysfunction in depression and cannabis use, the inclusion of EBEs was crucial in co-designing AMP-C to better understand their wants and needs. In this study, we detail the recruitment for a sample with co-occurring depressive symptoms and cannabis use, and we provide a framework for conducting a mixed methods interview with EBEs. Moreover, we implemented a mixed methods design by collecting both qualitative feedback and quantitative ratings of the intervention components from EBEs. These responses resulted in refinement of the intervention component and app design to better suit the preferences and needs of those with depressive symptoms and cannabis use. Thus, this work emphasizes how co-designing a digital intervention with EBEs can enhance the relevance of the digital intervention to better improve its engagement and fit for patients. Moreover, the current approach with EBEs yields important insight into how a scalable and self-guided digital intervention can target 2 problems or disorders simultaneously by taking a transdiagnostic approach. Future work will continue to refine AMP-C to improve it further so that it may be implemented as an alternative to in-person interventions.

## Appendix

Multimedia Appendix 1 COREQ (COnsolidated criteria for REporting Qualitative research) Checklist and the interview script for this study.

## Abbreviations

AMP: Amplification of Positivity
AMP-A: Amplification of Positivity for Alcohol Use Disorder
AMP-C: Amplification of Positivity—Cannabis Use Disorder
CBT: cognitive behavioral therapy
COREQ: COnsolidated criteria for REporting Qualitative research
C-SSRS: Columbia-Suicide Severity Rating Scale
CUD: cannabis use disorder
CUDIT-R: Cannabis Use Disorder Identification Test—Revised
EBE: experts by experience
MDD: major depressive disorder
NVS: negative valence system
PHQ-9: Patient Health Questionnaire-9
PVS: positive valence systems
RA: research assistant
SUD: substance use disorder

## Data Availability

The data that support the findings of this study are available on request from the corresponding author. The data are not publicly available due to the data-containing information that could compromise the confidentiality of research responses.
