# Supplementary material for: Inclusion of Individuals With Lived Experiences in the Development of a Digital Intervention for Co-Occurring Depression and Cannabis Use: Mixed Methods Investigation
Source: JMIR Form Res. 2024 Oct 7;8:e54751. doi: 10.2196/54751 (PMC11514326; doi:10.2196/54751)
Supplement: Multimedia Appendix 1 [file formative_v8i1e54751_app1.docx]

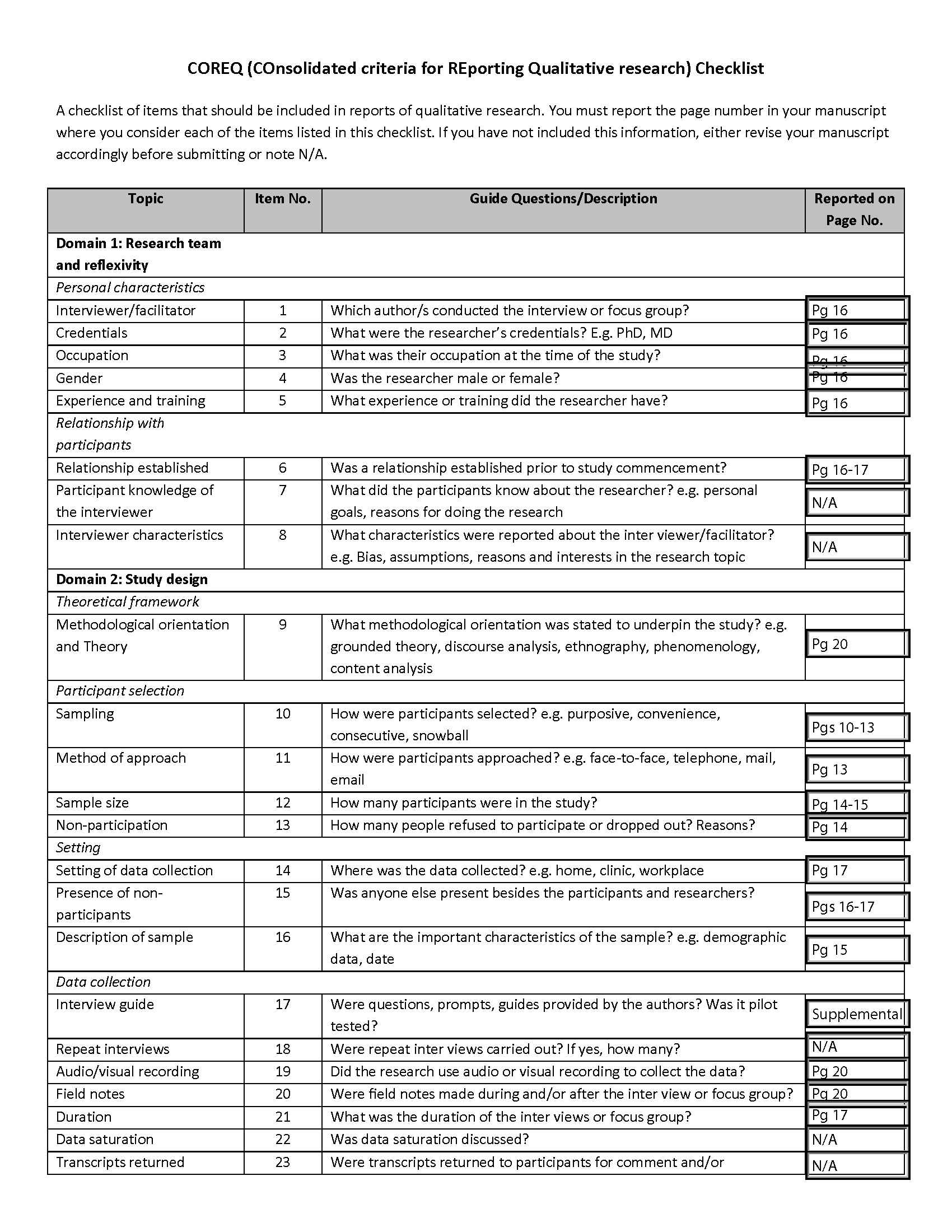

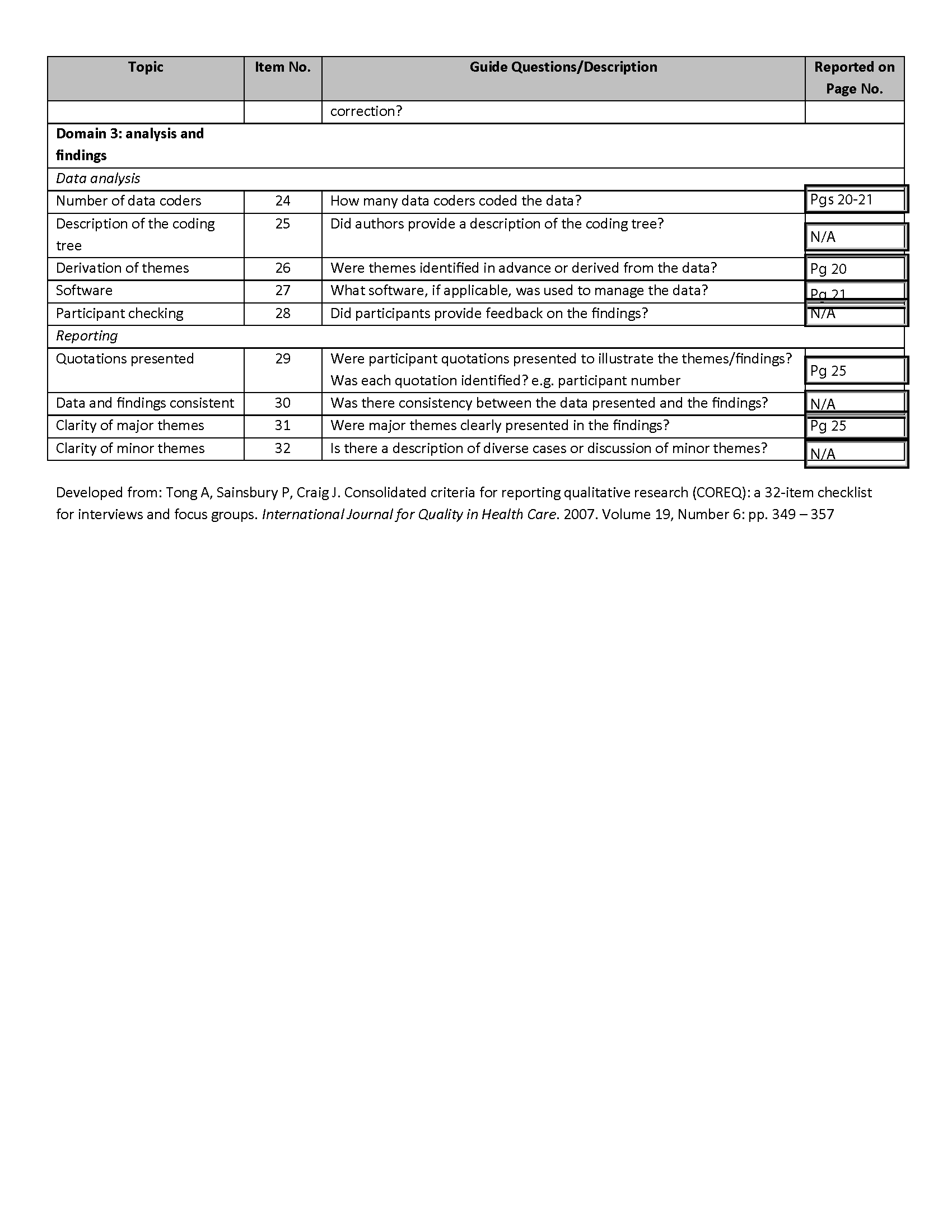


**Interview Script**

Thank you for participating in our research study. My name is _______ and I will be conducting this interview with you today. I am now going to begin the audio recording of our interview.

This interview will take approximately one hour to complete, and you will be invited back twice over the next six months to participate in two other interviews. At the end of our interview today, we will schedule your second interview for three months out.

Do you have any questions about this?

Will you be able to attend both interviews, spaced approximately three months apart?

The purpose of this first interview is to gather your opinions for a digital intervention as an app that we are creating for depression and cannabis use. This app will focus on specific activities and skills that can be beneficial in increasing positive emotions, reducing negative emotions, and improving overall well-being. During the first part of the interview today, I will ask you some questions about yourself, your experience with mental health apps and your mental health history. During the second part of the interview, I will ask you questions about what you would be interested in for a digital intervention. You will answer questions about which therapeutic skills would be helpful to learn, and identify content, strategies, and app features that would improve engagement, and you provide feedback on the preferred length of content and duration of the intervention.

At the end of the study, we aim to develop a self-guided digital intervention that can be accessed on Android phones, which may help individuals with depression and cannabis use in the future. Thus, the feedback that we gather from you during these interviews will be very helpful in creating this digital intervention.

Do you have any questions before we begin?

I will first ask you questions about yourself and your mental health history. All of your responses will be kept confidential, and you may skip any questions during this interview that you are not comfortable with answering.

- I will first gather demographic information from you.
- Can you provide me your first, middle, and last name as given to you at birth?
- What was your sex at birth?
- What was your date of birth?
- What city and state were you born in?

What electronic devices do you regularly use (e.g., a smartphone, laptop, tablet, smartwatch)

- How many hours per day do you estimate actively using your smartphone?
- What do you use your smartphone for?
- **Give following prompts as needed*
  - Do you use your smartphone for __________ (texting, calls, internet browsing, email)
  - What kind of apps do you use on your smartphone (e.g., social media, fitness, work, podcasts, etc.)?

Have you ever used mental health apps, such as Calm or Headspace?

- Which ones have you used?
- What features did you like about the app?
- What features did you dislike?
- Do you still use any mental health apps?
  - How often do you use it?
- If not, why did you stop using it?
- Overall, did you think ______ was helpful?
- What was helpful?
- What was not helpful?
- Is there any other information that you would like to share about the mental health apps that you have used ?

Have you ever seen somebody for emotional or psychiatric problems?

- What was that for?
- What type of treatment (therapy or medications)?
- How long did you attend?
- When did you first attend?
- When did you last attend?
- Did you think that experience was helpful?
- What aspects of therapy were helpful?
- What did you dislike?
- Are there any skills you learned in previous therapy that you still use?
  - What skills do you still use?

Have you ever attended a self-help group, like alcoholic anonymous, marijuana anonymous, or narcotics anonymous?

- Which one?
- How long or often did you attend?
- When did you first attend?
- When did you last attend?

What is your primary reason for using cannabis (for example, to relax, socially, or as a coping mechanism)?

- How do you use cannabis (e.g., edibles, smoking, etc.)?
- How much cannabis do you use on a given day?
- How often do you use cannabis?
- How long have you been using cannabis?

Would you engage with a mental health app for depressive symptoms and cannabis use?

- If you were to engage with a mental health app for depressive symptoms and cannabis use, what would you like to have included?
- How would you picture yourself fitting a mental health app into your current daily routine or lifestyle?
- How often would you want to engage with the app?
- How many minutes would you like to engage with each content at a time?
- How often would you want to learn a new skill through the app (for example, every day, once a week, etc.)?

Would you find it helpful to have reminders/methods to keep you accountable for participating in a mental health app?

- If yes, what would be helpful? *(Examples for prompting: push notifications, engagement activities, text or email reminders)*

Would you prefer to be able to use it more passively (i.e., reading things while sitting down/watching a video) or actively (i.e., a breathing exercise or guided meditation/mindfulness)?

- Would you like to have tasks to complete in between content to help practice your skills? These could include, for example, answering short questions about your skill practices.
- Would you like to be able to track your symptoms over time in the app?

How would you like the content presented to you? For example, would you prefer that everything is available to you at once, with recommendations of content to complete first? Or would you prefer a structured sequence where you get access to each content at a time?

Would you be interested in receiving bonus or advanced content ? For example, if you learned about and practiced one skill, you would then have the opportunity for additional content where you could learn how to master that skill further.

Do you have any other suggestions or feedback that you would like to see in an app?

Now I am going to provide you with some scenarios regarding different content that we might include in the digital intervention. These activities and content are intended to increase positive emotions, thinking, and behavior and decrease your depressive symptoms and cannabis use. I would like your feedback about each of these, including giving your overall thoughts and rating each content:

Prior research has indicated a link between cannabis use and depression.

- What kind of educational content would you like to receive regarding cannabis use and depressive symptoms?

This content would include education about the pros and cons of cannabis use, including how it may impact your daily life, mood and behavior. You would then learn about making concrete goals for yourself and identify goals for changing your cannabis use.

- What are your thoughts about this content?
- On a scale from 1-5 (1 being not at all and 5 being extremely), how helpful do you think this content would be in terms of reducing your cannabis use and depressive symptoms?
- On a scale from 1-5 (1 being not at all and 5 being extremely), how interesting do you think this content would be to complete?
- Are there any other skills or content regarding cannabis use that you would like to have included?
- Is there any other feedback on this content that you would like to provide?

Our emotions, including positive and negative emotions, can impact our thinking, behaviors, and overall moods.

- What kind of educational content would you like regarding the role of positive and negative emotions?

You could learn about how negative emotions can increase urges to use cannabis or other substances and learn skills related to self-monitoring your mood, cannabis use, and cravings.

- What are your thoughts about this content?
- On a scale from 1-5, how helpful do you think this content would be in terms of reducing your cannabis use and depressive symptoms?

**Repeat 1-5 scale as needed*  (1 being not at all and 5 being extremely)

- On a scale from 1-5, how interesting do you think this content would be to complete?

**Repeat 1-5 scale as needed*  (1 being not at all and 5 being extremely)

- Are there any other skills or content related to emotions and self-monitoring that you would like to have included?
- Is there any other feedback on this potential content that you would like to provide?

Some individuals have difficulties experiencing positivity. Specifically, some people may not want to experience positive emotions, including happiness and joy, due to these emotions being linked to previous negative outcomes. For example, a previous, positive experience could have ended in disappointment.

- What kind of educational content would you like related to difficulties experiencing positivity?

You would also learn about how one’s views of positivity impacts their depressive symptoms and cannabis use.

- What are your thoughts about this content?
- On a scale from 1-5, how helpful do you think this content would be in terms of reducing your cannabis use and depressive symptoms ?
- On a scale from 1-5, how interesting do you think this content would be to complete?
- Are there any other skills or content related to difficulties experiencing positivity that you would like to have included?
- Is there any other feedback on this content that you would like to provide?

Identifying and noticing positive events in your life can be helpful in reducing depressive symptoms and cannabis use. Specifically, drawing your attention to positive events, rather than focusing on only negative events, can be helpful.

- What kind of educational content would you like related to increasing attention to positive events?

Bringing awareness to the positive events in our life and scheduling more positive activities can improve overall well-being and positive emotions. Thus, you can learn content and skills related to scheduling positive activities that do not involve cannabis so that you experience more positive emotions daily.

- What are your thoughts about this content?
- On a scale from 1-5, how helpful do you think this content would be in terms of reducing your cannabis use and depressive symptoms ?
- On a scale from 1-5, how interesting do you think this content would be to complete?
- Are there any other skills or content related to scheduling positive activities that you would like to have included?
- Is there any other feedback on this content that you would like to provide?

When we do experience positive emotions, they can be fleeting or temporary. So, it can be helpful to learn how to prolong, amplify, or intensify our positive emotions.

- What kind of educational content would you like related to increasing prolonging positive emotions?

One way to extend the positive emotions following positive events is to capitalize on your experience in the moment. Specifically, savoring, telling someone about it, writing about it, revisiting it in your mind, or re-experiencing the positive emotions, can be helpful ways to extend the positive events or intensify the positive emotions that you experience.

- What are your thoughts about this content?
- On a scale from 1-5, how helpful do you think this content would be in terms of reducing your cannabis use and depressive symptoms ?
- On a scale from 1-5, how interesting do you think this content would be to complete?
- Are there any other skills or content related to prolonging positive emotions that you would like to have included?
- Is there any other feedback on this content that you would like to provide?

Gratitude, or showing thankfulness or appreciation for the different people or things in your life, can help to improve positive emotions.

- What kind of educational content would you like related to gratitude?

Writing about and reflecting on things that you are grateful for is another way that you can increase your attention to positivity in your life. Thus, it could be helpful to learn specific skills of how you could practice gratitude in your daily life.

- What are your thoughts about this content?
- On a scale from 1-5, how helpful do you think this content would be in terms of reducing your cannabis use and depressive symptoms ?
- On a scale from 1-5, how interesting do you think this content would be to complete?
- Are there any other skills or content related to gratitude that you would like to have included?
- Is there any other feedback on this content that you would like to provide?

Research has shown that completing acts of kindness, or doing things for others, can help improve your mood.

- What kind of educational content would you like related to acts of kindness?

Engaging in different acts of kindness, including for family, friends, and strangers, can benefit your well-being and positive emotions. So, it may be helpful to engage in different acts of kindness in your daily life.

- What are your thoughts about this content?
- On a scale from 1-5, how helpful do you think this content would be in terms of reducing your cannabis use and depressive symptoms ?
- On a scale from 1-5, how interesting do you think this content would be to complete?
- Are there any other skills or content related to acts of kindness that you would like to have included?
- Is there any other feedback on this content that you would like to provide?

There are three different types of activities, including pleasurable, engaging, and meaningful activities. Each type of activity can create a different experience and emotion, which overall increases positive emotions.

- What kind of educational content would you like related to these three different activities?

Learning about the difference between activities that are pleasurable (enjoyable), engaging (fully immersing), and meaningful (important) is important for recognizing how we can complete a variety of different, positive activities in our lives. Thus, you could receive content focusing on engaging in these different activities to improve your mood and symptoms.

- What are your thoughts about this content?
- On a scale from 1-5, how helpful do you think this content would be in terms of reducing your cannabis use and depressive symptoms ?
- On a scale from 1-5, how interesting do you think this content would be to complete?
- Are there any other skills or content related to pleasurable, engaging, and meaningful activities that you would like to have included?
- Is there any other feedback on this content that you would like to provide?

Engaging in activities that are in line with your values and strengths can improve your positive views about yourself and your world.

- What kind of educational content would you like related to values and strengths?

You could work to identify the things that you value in life, including things that make life worthwhile, and learn how to engage in activities that are consistent with those values. You could also learn to recognize your strengths and identify ways to use those strengths in your daily life.

- What are your thoughts about this content?
- On a scale from 1-5, how helpful do you think this content would be in terms of reducing your cannabis use and depressive symptoms ?
- On a scale from 1-5, how interesting do you think this content would be to complete?
- Are there any other skills or content related to values and strengths that you would like to have included?
- Is there any other feedback on this content that you would like to provide?

There are several benefits to optimism, or having hope and confidence about the future.

- What kind of educational content would you like related to optimism?

Imagining your best possible future can help you focus your attention towards the more positive aspects of yourself and your future. Building and cultivating optimism, can help train ourselves to take a different perspective of the world and shift our lens to considering more positive future outcomes. Thus, you could learn skills to help imaging your best possible future and writing about it to increase your positive emotions and well-being.

- What are your thoughts about this content?
- On a scale from 1-5, how helpful do you think this content would be in terms of reducing your cannabis use and depressive symptoms ?
- On a scale from 1-5, how interesting do you think this content would be to complete?
- Are there any other skills or content related to optimism that you would like to have included?
- Is there any other feedback on this content that you would like to provide?

One of the most consistent findings in psychological research is that social relationships are important for positive mental health and well-being.

- What kind of educational content would you like related to social relationships?

Focusing your attention towards doing positive things for others can reduce negative thoughts and emotions that come with depression. Learning to help make someone else experience positive emotions may also make you experience positive emotions. This content could help you identify and develop ways that you can make someone else happier.

- What are your thoughts about this content?
- On a scale from 1-5, how helpful do you think this content would be in terms of reducing your cannabis use and depressive symptoms ?
- On a scale from 1-5, how interesting do you think this content would be to complete?
- Are there any other skills or content related to social relationships that you would like to have included?
- Is there any other feedback on this content that you would like to provide?

Learning to “seize the day” and live this month like it will be your last in this area can help focus your attention on things that you are grateful for and increase positive emotions.

- What kind of educational content would you like related to living your month like it is your last in the area?

Developing skills to identify and engage in activities that are in line with your values about where you live now can also increase your engagement in pleasurable and meaningful activities. Thus, you will learn strategies to live the next month to the fullest and increase your positive emotions and thoughts.

- What are your thoughts about this content?
- On a scale from 1-5, how helpful do you think this content would be in terms of reducing your cannabis use and depressive symptoms ?
- On a scale from 1-5, how interesting do you think this content would be to complete?
- Are there any other skills or content related to living your month like it is your last in the area that you would like to have included?
- Is there any other feedback on this content that you would like to provide?

Although the different content that I just described to you can be helpful for many people, not all of this content may be specifically helpful to you and improve your positive mood. Indeed, you may prefer certain content to others that you learn in this intervention. So, it’s important to individualize the skills for you and make plans to implement them in your daily life moving forward.

- What kind of educational content would you like related to making individualized plans from this content to fit your interests and needs?

You would identify the different content that is most helpful for you. Then, you would learn how to develop a personalized treatment plan so that you can continue to experience sustainable gains in positive emotions and prevent relapse of cannabis use and depressive symptoms.

- What are your thoughts about this content?
- On a scale from 1-5, how helpful do you think this content would be in terms of reducing your cannabis use and depressive symptoms ?
- On a scale from 1-5, how interesting do you think this content would be to complete?
- Are there any other skills or content related to making individualized plans that you would like to have included?
- Is there any other feedback on this content that you would like to provide?

This concludes our interview for today. Do you have any other questions or feedback to provide?

The next steps will include scheduling your second interview over Zoom, which will be approximately three months from now. We will schedule this with you now and periodically follow up with you to determine if the time scheduled still works for you. During this second interview, we will show you a prototype or mock-up of the intervention, including any videos that we created. We will also ask for your opinions and feedback regarding the intervention materials.

[count 3 months from today]. Does ________ at ________am/pm work for you?

Thank you for scheduling your second interview. Another researcher will reach out to you over the next week to confirm your mailing address and mail your $50 Amazon giftcard for completing this qualitative interview. Do you have any questions before we end today?
